# Generation of tumor antigen-specific CD4+ and CD8+ T cells by simultaneous MHC-I and -II epitope presentation in vitro and *in vivo*

**DOI:** 10.1186/2051-1426-2-S3-P65

**Published:** 2014-11-06

**Authors:** Carina Wehner, Christian Ellinger, Silke Raffegerst, Susanne Wilde, Barbara Mosetter, Judith Eckl, Bernhard Frankenberger, Manon Weis, Giulia Longinotti, Nadja Sailer, Dolores J Schendel, Slavoljub Milosevic

**Affiliations:** 1 Trianta Immunotherapies GmbH - a subsidiary of Medigene AG, Germany; 2 Institute of Molecular Immunology/Helmholtz Center Munich, Germany

## 

In recent years, activation of the patient's immune system to defend against tumors was demonstrated to be a promising alternative strategy to classical cancer treatments. Dendritic cells (DCs) can present antigens on MHC-II and -I leading to the activation of CD4^+ ^or CD8^+ ^T cells, respectively. Stimulated CD4^+ ^T cells act as helper cells for cytotoxic CD8^+ ^T cells to kill tumor cells by mediating strong proliferation and licensing DCs, increasing their presentation capacity. Tumors bearing MHC-II molecules can also be directly destroyed by cytotoxic CD4^+ ^T cells. As an efficient anti-tumor response strongly depends on the interplay of DCs, CD4^+ ^and CD8^+ ^T cells, these three cell types are considered to be essential for successful immunotherapy design.

In clinical trials, DCs were either endogenously or exogenously loaded with tumor antigens for the presentation on MHC-I rather MHC-II. As the antigen presentation pathways differ, a simultaneous loading of MHC-I and -II was suboptimal. To overcome this obstacle, we used a signaling sequence (CrossTAg) to force MHC-II cross-presentation of tumor-associated antigens (TAAs) encoded by *in vitro *transcribed (*ivt*) RNA. Subsequently, peripheral blood lymphocytes were primed by DCs expressing TAAs on MHC-I and -II enabling activation and interaction of CD4^+ ^and CD8^+ ^T cells. By this approach, we were able to induce and identify TAA-specific CD4^+ ^and CD8^+ ^T cells in the same experiment.

In addition, superior induction efficiency of DCs loaded with CrossTAg-TAA-*ivt*RNA compared to conventional TAA-*ivt*RNA was shown in the humanized NOD/scid IL2Rgnull (NSG) mouse model. NSG mice were engrafted with human peripheral blood mononuclear cells (PBMC) and vaccinated twice with DCs either transfected with CrossTAg-TAA-*ivt*RNA or conventional TAA-*ivt*RNA. Reisolated PBMC of mice vaccinated with CrossTAg-TAA-*ivt*RNA transfected DCs showed higher numbers of antigen-specific CD8^+ ^T cells and stronger activation/cytotoxic activity against tumor cells.

These in vitro and in vivo data illustrate the benefits of loading DCs with CrossTAg-linked target antigens as CD8^+ ^and CD4^+ ^T cells can be induced side by side allowing interactions and T cell help.

